# Oncologic and survival outcomes of pT2N0 rectal adenocarcinoma treated by transanal local excision: a retrospective cohort study

**DOI:** 10.1007/s00464-025-12239-6

**Published:** 2025-09-29

**Authors:** Alberto Arezzo, Carlo Alberto Ammirati, Giovanni Distefano, Michele Barbiero, Roberto Passera, Mario Morino

**Affiliations:** 1 Department of Surgical Sciences, University of Turin, Città della Salute e della Scienza Hospital, Turin, Italy; 2 Division of Nuclear Medicine, University of Turin, Città della Salute e della Scienza Hospital, Turin, Italy

**Keywords:** pT2 rectal cancer, Transanal excision, Local recurrence, Radiotherapy, Salvage surgery

## Abstract

**Background:**

The optimal management of pT2N0 rectal adenocarcinoma remains controversial, especially when tumours are incidentally diagnosed after local excision. Although total mesorectal excision (TME) is the standard approach, its associated morbidity has led to the exploration of conservative strategies. This study compares oncologic outcomes amongst three post-excision management options: salvage surgery, adjuvant radiotherapy, and no further treatment.

**Methods:**

This retrospective cohort comprised 90 patients with pT2N0 rectal adenocarcinoma who were treated by transanal excision at a single tertiary centre from 1993 to 2025. All patients were staged N0 on MRI and divided into three groups: Group A (no further treatment), Group B (adjuvant radiotherapy), and Group C (completion salvage surgery). The primary outcomes were overall survival (OS) and disease-free survival (DFS), with additional analyses of histopathologic prognostic factors.

**Results:**

Median follow-up was 31 months. OS varied significantly across groups (p = 0.015), with the highest survival in Group B, followed by Group C and Group A. DFS showed no significant difference between groups. Patients in Group B had the lowest mortality (8.7%) and recurrence (30.4%) rates. Tumour budding was a significant predictor of poor prognosis in multivariate analysis. Local excision alone was associated with higher recurrence and mortality rates.

**Conclusion:**

In selected patients with pT2N0 rectal cancer discovered after transanal excision, adjuvant radiotherapy may provide outcomes comparable to salvage surgery and serve as an alternative in patients not suitable for surgery. High-risk histopathologic features should inform further management, as local excision alone was associated with worse outcomes. Multidisciplinary evaluation remains crucial for treatment planning.

The management of pT2N0 rectal adenocarcinoma presents an enduring clinical dilemma: how to balance the oncologic robustness of radical surgery with the functional preservation offered by less invasive approaches. Total mesorectal excision (TME) remains the standard for oncologic treatment, providing reliable local control and long-term survival. However, TME carries significant functional costs, including urinary and sexual dysfunction, anterior resection syndrome, and in many cases, temporary or permanent stoma formation, all of which substantially affect patients’ quality of life. These limitations emphasise the growing interest in alternative strategies that offer curative potential whilst preserving anorectal and pelvic function.

In this context, transanal endoscopic microsurgery (TEM) has increasingly gained attention as a minimally invasive, organ-preserving option. Whilst initially developed for early stage tumours, its use in pT2 lesions, particularly when combined with neoadjuvant chemoradiotherapy (CRT), has shown promising oncological outcomes. Buess et al. first reported a 93% cancer-specific survival and a 13% local recurrence rate at a median follow-up of 255 months in patients with pT2 disease treated with CRT-TEM [1]. Similarly, Duek et al. demonstrated that adjuvant radiotherapy following TEM significantly reduced local recurrence and improved disease-free survival (DFS) in selected high-risk cases [2].

A crucial milestone was achieved with the randomized controlled trial by Lezoche et al. [3], which was the first to compare neoadjuvant radiotherapy plus local excision against standard TME in patients with cT2N0 rectal cancer. After 84 months of follow-up, the study demonstrated equivalent DFS and only marginal differences in local recurrence rates (5.7% vs 2.8%), establishing a basis for subsequent structured evaluations of transanal strategies within oncological protocols. In the years since, numerous studies have replicated these promising findings, especially in well-selected patients who respond favourably to neoadjuvant CRT and lack adverse pathological features.

In contrast, the role of postoperative radiotherapy after local excision has been more debated. Early data were inconsistent, and the absence of standardised protocols created uncertainty about its true oncological benefit. Furthermore, the risk of undertreatment in patients with high-risk histological features—such as lymphovascular invasion, poor differentiation, or tumour budding—has restricted its widespread use. As a result, salvage TME remains the standard recommendation in these cases, providing more consistent oncological outcomes when adverse features are identified after local excision.

The therapeutic landscape has advanced further with the introduction of transanal minimally invasive surgery (TAMIS) approximately 15 years ago. TAMIS has overcome several technical barriers associated with TEM—such as limited instrumentation and rectal access—allowing for wider adoption of transanal excision, particularly within organ-preserving treatment strategies. It has also reignited interest in combining local excision with neoadjuvant or adjuvant therapies to replicate the outcomes of radical surgery without its associated morbidity.

Against this backdrop, patient selection remains the key challenge. Identifying which patients with incidental pT2N0 tumours found after local excision can safely forgo salvage TME in favour of adjuvant radiotherapy or surveillance is crucial to expanding organ preservation without compromising oncological safety.

This study aims to assess oncological outcomes, postoperative complications, and functional preservation in patients with pT2N0 rectal adenocarcinoma identified after local excision (TEM), presumed to be node-negative based on imaging results. We compare three real-world management approaches—completion salvage surgery, adjuvant radiotherapy, or no further treatment—to refine selection criteria and guide clinical decisions in this increasingly complex therapeutic landscape.

## Material and methods

### Study design and patient population

This is a retrospective observational study including patients with histologically confirmed pT2 rectal adenocarcinoma, assumed to be N0 based on imaging, who underwent transanal excision between 1993 and 2025 at a single tertiary referral centre. All patients were treated initially with local excision via transanal endoscopic microsurgery (TEM) or equivalent minimally invasive transanal technique. Patients were included if they had:

- Final pathology confirming pT2 tumour stage
- Negative lymph node status (N0) at MRI
- No distant metastasis at the time of diagnosis
- Complete follow-up data available for survival analysis

Patients treated for benign disease, or for tumour stages other than pT2N0, were excluded.

### Data collection

Demographic, clinical, surgical, histopathological, and oncologic follow-up data were retrieved from a prospectively maintained institutional database. The following variables were collected and analysed:

- Demographics: age, gender, date of surgery, date of birth
- Tumour characteristics: tumour distance from the anal verge, tumour diameter, tumour grading, tumour budding, lymphovascular invasion, and perineural invasion
- Staging tools used: endorectal ultrasound (EUS) and pelvic MRI for both tumour invasion and nodal assessments
- Margins: quality and clearance, including circumferential resection margin where applicable
- Outcomes: date and type of recurrence (local or distant), date of death

### Definition of patient subgroups

A key stratification variable was the management of patients concerning salvage surgery after local excision, defined as any surgical procedure performed after a non-curative or failed local treatment, aiming at removing residual or recurrent cancerous rectal tissue. Patients were divided into three predefined groups:

- Group A—Observation: for patients’ refusal to salvage surgery and adjuvant therapy, or because they are judged unfit for both
- Group B—Adjuvant Radiotherapy: for patients’ refusal to electively salvage surgery (within 2 months after TEM) or because they are judged unfit for it based on a multidisciplinary team decision; all patients were administered long-course RT, without chemotherapy**.**
- Group C—Salvage Surgery: patients who underwent completion radical surgery (TME) electively (within 2 months after TEM) following local excision.

### Outcomes

The primary study outcomes were:

- Overall Survival (OS) defined as the time from the date of transanal surgery to death from any cause.
- Disease-Free Survival (DFS) defined as the time from surgery to the first event of local recurrence, distant metastasis, or death related to rectal cancer.

Secondary outcomes included the rate and timing of local recurrence, as well as the identification of histopathologic predictors of adverse outcomes.

### Statistical analysis

Survival curves were estimated by the Kaplan–Meier method and compared across groups by the log-rank test. The effect on OS and DFS of the same set of risk factors has been estimated using univariate and multivariate Cox proportional hazards regression models, comparing the two arms with the Wald test and calculating 95% confidence intervals. Patient characteristics were analysed using Fisher’s exact test for categorical variables and the Mann–Whitney and Kruskal–Wallis tests for continuous variables; continuous covariates were described as median/Inter Quartile Range (IQR). All reported p-values were obtained by the two-sided exact method at the conventional 5% significance level.

Stratified analyses were conducted for the three groups (Groups A, B, and C) and histopathologic risk factors, including tumour grading, tumour budding, lymphovascular invasion, perineural invasion, and tumour size. Tumour diameter was analysed both as a continuous variable and dichotomised using the median value. A multivariable Cox proportional hazards model was planned to identify independent predictors of OS and DFS. Statistical significance was set at *p* < 0.05.

Data were analysed as of July 2025 by R 4.5.1 (R Foundation for Statistical Computing, Vienna-A, http://www.R-project.org).

## Results

### Patient population and clinicopathological characteristics

A total of 90 patients with histologically confirmed pT2 rectal adenocarcinoma, assumed to be N0 based on imaging, who underwent transanal local excision by TEM between 1993 and 2025, were included in the present analysis. Based on subsequent therapeutic strategies, the cohort was stratified into three groups: 40 (44.4%) received no further treatment (Group A), 23 (25.6%) underwent postoperative radiotherapy (Group B), and 27 patients (30.0%) underwent completion salvage surgery (Group C). All postoperative radiotherapies were started and salvage surgeries performed between 8 and 10 weeks after index local excision.

The median age at diagnosis was 72 years (interquartile range [IQR], 64–80), and 42.2% of patients were female. The majority of tumours were classified as well-differentiated tumours (G1) in 10.0% of cases, moderately differentiated (G2) in 75.6%, whilst poorly differentiated (G3) in 14.4%, respectively. High-grade tumour budding was reported in 14.4% of patients, and mucinous histology in 16.7% of the cases. Lymphovascular invasion was detected in 12.2%, and perineural invasion in 5.6% of the specimens. The median tumour diameter was 3.0 cm (IQR, 2.0–4.0 cm). The median distance from the anal verge was 6.0 cm (IQR, 5.0–8.0 cm). Margins were positive in 13 (14.4%) of patients, with an equal distribution amongst the Groups (p = 0.755). The median duration of follow-up for the entire cohort was 31 months (interquartile range, IQR, 16–52).

Baseline clinicopathological characteristics were relatively well balanced across treatment groups. However, patients in the salvage surgery group tended to be younger and marginally more likely to exhibit adverse histological features, including lymphovascular and perineural invasion (Table 1).

**Table 1:** Summary of patient characteristics

Variable	Overall	Salvage surgery	No further treatment	Post-op radiotherapy	*p*-value
	*n* =9 0	(*n* = 27)	(*n* = 40)	(*n* = 23)	
Age (median)	72 (64–80)	68 (57–72)	77 (69–84)	71 (64–77)	0.001
Gender					0.028
Male	52 (57.8%)	11 (40.7%)	23 (57.5%)	18 (78.3%)	
Female	38 (42.2%)	16 (59.3%)	17 (42.5%)	5 (21.7%)	
Grading					0.758
G1	9 (10.0%)	2 (7.4%)	3 (7.5%)	4 (17.4%)	
G2	68 (75.6%)	21 (77.8%)	31 (77.5%)	16 (69.6%)	
G3	13 (14.4%)	4 (14.8%)	6 (15.0%)	3 (13.0%)	
Mucinous type					0.685
Absent	74 (82.2%)	21 (77.8%)	34 (85.0%)	19 (82.6%)	
Present	15 (16.7%)	6 (22.2%)	5 (12.5%)	4 (17.4%)	
N/A	1 (1.1%)	0 (0.0%)	1 (2.5%)	0 (0.0%)	
Tumour budding					0.064
Low-grade	77 (85.6%)	21 (77.8%)	33 (82.5%)	23 (100.0%)	
High-grade	13 (14.4%)	6 (22.2%)	7 (17.5%)	0 (0.0%)	
Lymphovascular invasion					0.746
Absent	79 (87.8%)	24 (88.9%)	34 (85.0%)	21 (91.3%)	
Present	11 (12.2%)	3 (11.1%)	6 (15.0%)	2 (8.7%)	
Perineural invasion					0.403
Absent	85 (94.4%)	25 (92.6%)	37 (92.5%)	23 (100.0%)	
Present	5 (5.6%)	2 (7.4%)	3 (7.5%)	0 (0.0%)	
Tumour diameter (cm, median)	3 (2–4)	3 (2–4)	3 (3–4)	3 (2–3)	0.086
Distance from the anal verge (cm, median)	6 (5–8)	6 (4-8)	6 (5–7)	8 (5–9)	0.404
Margins					0.755
>1mm	77 (85.6%)	24 (88.9%)	34 (85.0%)	19 (82.6%)	
<1mm	13 (14.4%)	3 (11.1%)	6 (15.0%)	4 (17.4%)	
ASA score					0.758
1	9 (10.0%)	2 (7.4%)	3 (7.5%)	4 (17.4%)	
2	66 (73.3%)	21 (77.8%)	29 (72.5%)	16 (69.6%)	
3	13 (14.4%)	4 (14.8%)	6 (15.0%)	3 (13.0%)	
4	2 (2.3%)	0 (14.8%)	2 (5.0%)	0 (13.0%)	

### Survival outcomes by treatment group

**Fig. 1:**
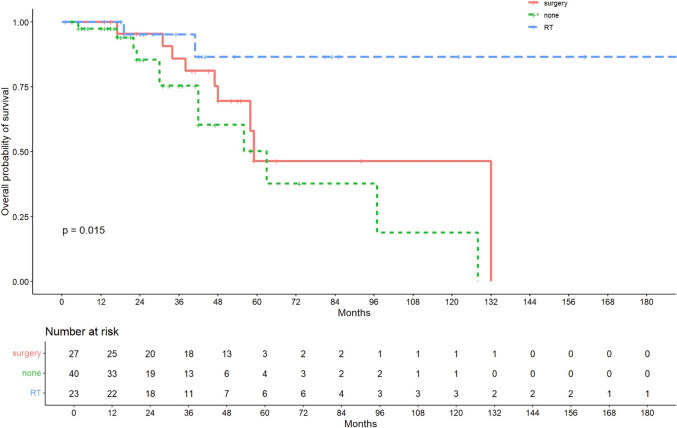
Survival curves estimated by the Kaplan–Meier method for Overall Survival (OS) divided by groups (Group A (no further treatment), Group B (adjuvant radiotherapy), and Group C (completion salvage surgery))

**Fig. 2:**
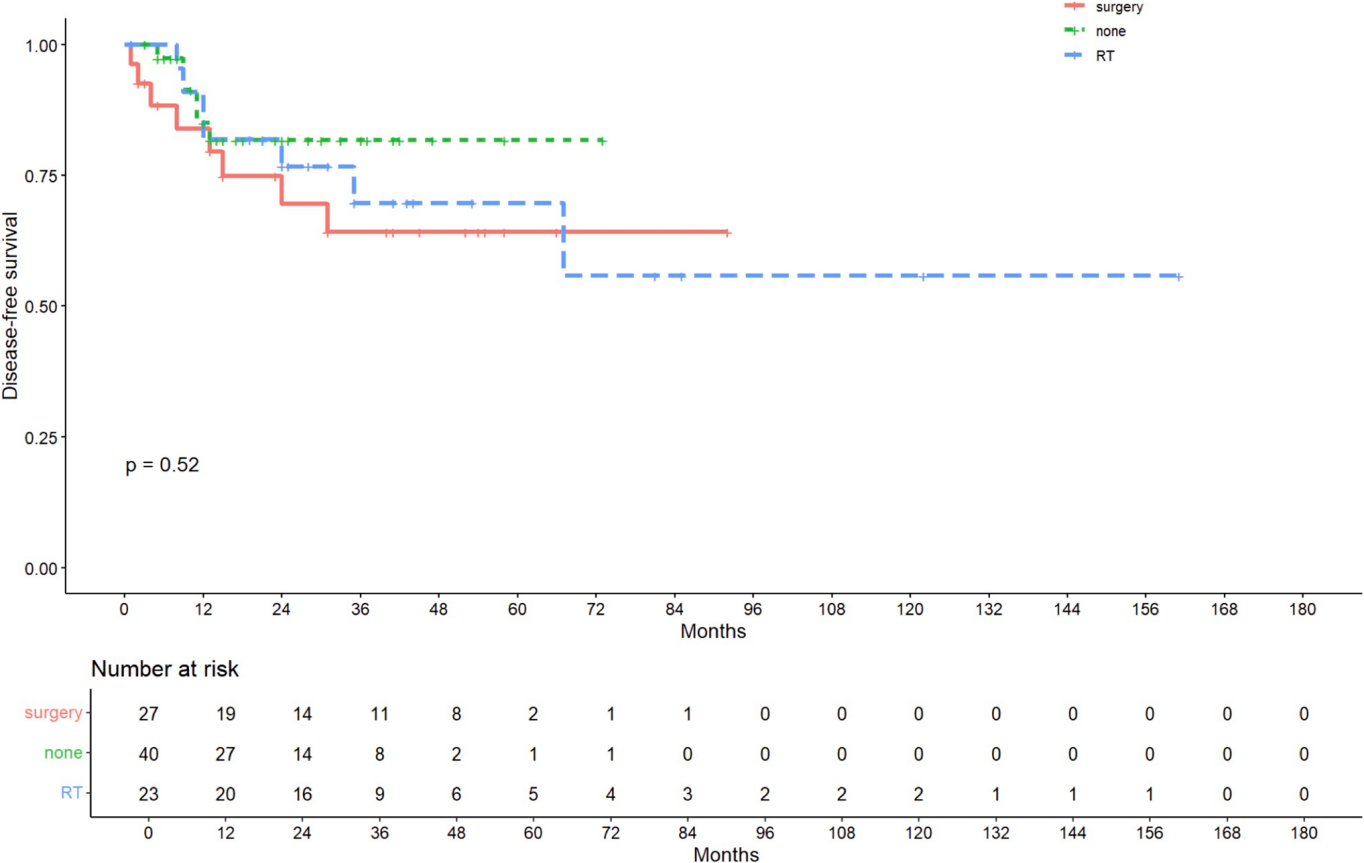
Survival curves estimated by the Kaplan–Meier method for Disease-free Survival (DFS) divided by groups (Group A (no further treatment), Group B (adjuvant radiotherapy), and Group C (completion salvage surgery))

At the time of the final follow-up, 23 patients (25.6%) had died and 21 (23.3%) had experienced disease recurrence. As shown in Figure 1, overall survival (OS) was longest in patients who underwent postoperative radiotherapy (group B), followed by the salvage surgery group (group C), and shortest in the group managed with local excision alone (group A), showing a statistically significant difference across the three treatment strategies (p = 0.015). Similarly, disease-free survival (DFS) was most favourable in the radiotherapy group (group B), followed by no further treatment (group A) and salvage surgery (group C), as highlighted in Figure 2, but unlike OS, did not show any statistically significant difference amongst the three groups. The marginal protective role of RT was confirmed in the OS univariate and multivariate models, as well as in the DFS univariate model.

Mortality was highest amongst patients who received no further treatment (group A: 12/40, 30.0%), compared with 9/27 (33.3%) in the salvage surgery group (group C) and 2/23 (8.7%) in the radiotherapy group (group B). At overall survival (OS), univariate analysis revealed that disease recurrence occurred in 29.6% of patients undergoing salvage surgery (group C: 8/27), 30.4% of those receiving postoperative radiotherapy (group B: 7/23), and 15.0% of patients in the no-treatment group (group A: 6/40), as summarised.

### Prognostic impact of clinicopathological variables

The Cox proportional hazard regression models for OS and DFS are shown in Table 2 and Table 3, respectively.

**Table 2:** Cox proportional hazard regression models for OS

	Univariate		Multivariate	
	HR (95% CI)	*p*	HR (95% CI)	*p*
Treatment		0.035		0.028
None vs surgery	1.64 (0.67–4.00)	0.275	1.95 (0.76–5.01)	0.166
RT vs surgery	0.22 (0.05–1.04)	0.057	0.26 (0.05–1.25)	0.093
Tumour budding (high vs low grade)	1.66 (0.61–4.51)	0.324	-	-
Lymph Vascular invasion (yes vs no)	1.52 (0.45–5.21)	0.502	-	-
Perineural invasion (yes vs no)	4.33 (0.97–19.28)	0.055	4.56 (0.93–22.43)	0.062
Mucinous type (yes vs no)	0.65 (0.15–2.80)	0.562	-	-
Tumour grading (G3 vs G1+G2)	1.76 (0.51–6.09)	0.370	-	-

**Table 3:** Cox proportional hazard regression models for DFS

	Univariate		Multivariate	
	HR (95% CI)	*p*	HR (95% CI)	*p*
Treatment		0.531	-	-
None vs surgery	0.54 (0.19–1.57)	0.261	-	-
RT vs surgery	0.78 (0.28–2.19)	0.638	-	-
Tumour budding (high vs low grade)	3.98 (1.52–10.47)	0.005	4.14 (1.57–10.95)	0.004
Lymph Vascular invasion (yes vs no)	1.49 (0.44–5.08)	0.521	-	-
Perineural invasion (yes vs no)	3.79 (0.85–16.85)	0.080	2.38 (0.50–11.40)	0.277
Mucinous type (yes vs no)	2.36 (0.91–6.11)	0.077	2.50 (0.96–6.47)	0.062
Tumour grading (G3 vs G1+G2)	1.94 (0.71–5.28)	0.198	-	-

Amongst patients with lymphovascular invasion, the mortality rate was higher (3/11, 27.3%) compared to those without lymphovascular involvement (20/79, 25.3%), data confirmed by univariate OS and DFS, but without statistical significance (OS univariate HR 1.52, 95% CI 0.45–5.21, p 0.502 and DFS univariate HR 1.49, 95% CI 0.44–5.08, p 0.521). High-grade tumour budding, present in 13 patients, was associated with increased recurrence (6/13, 46.2%) and mortality (5/13, 38.5%), with a significant difference in DFS univariate (HR 3.98, 95% CI 1.52–10.47, p 0.005) and multivariate models (HR 4.14, 95% CI 1.57–10.95, p 0.004). High tumour grade at histology (G3), compared to low and moderate (G1–G2), showed an increased risk of death (3/13, 23.1%) and recurrence (5/13, 38.5%), although these results were not statistically significant at OS and DFS univariate analysis.

Although limited by the small sample size, patients with perineural invasion demonstrated the poorest outcomes, with 2 of 5 patients (40.0%) dying during follow-up and 2 experiencing recurrence. This trend played a marginal role in the OS univariate model (HR 4.33, 95% CI 0.97–19.28, p = 0.055) and reached significance in multivariate analysis (HR 10.08, 95% CI 1.87–54.25, p = 0.007). The mortality rate for patients diagnosed with mucinous tumours was 13.3% (2/15), with a recurrence rate of 40% (6/15), showing a tendency towards significance in DFS univariate analysis (HR 2.36, 95% CI 0.91–6.11, p 0.077) and multivariate analysis (HR 2.50, 95% CI 0.96–6.47, p 0.062). Tumour diameter also appeared to influence survival when exceeding 3 cm, with a mortality rate of 33.3% (11/33) and a recurrence rate of 24.2% (8/33).

## Discussion

The optimal management of pT2N0 rectal adenocarcinoma remains a topic of ongoing clinical debate [4]. Whilst total mesorectal excision (TME) has traditionally been regarded as the standard for oncological control, its significant functional morbidity has led to increasing interest in less invasive options, such as transanal local excision followed by adjuvant therapy in selected patients. Our study provides real-world evidence on outcomes after local excision, comparing subsequent approaches—namely salvage surgery, adjuvant radiotherapy, or observation alone—and their effects on survival.

Since the seminal trial by Lezoche et al. [3] in 2011, which demonstrated equivalent disease-free survival and only minor differences in local recurrence (5.7% vs 2.8%) at 84 months, several studies have confirmed these findings. The ACOSOG Z6041 phase II trial reported local and distant recurrence rates of 4% and 6%, respectively, at a median follow-up of 56 months [5]. The GRECCAR II randomized controlled trial further reinforced these results, comparing local excision with TME in patients with T2–T3 low rectal cancer, showing a favourable response to neoadjuvant CRT. It found no significant differences in oncologic outcomes between the two arms, supporting local excision as a potentially oncologically safe strategy in carefully selected patients with close follow-up [6]. More recently, the TAUTEM trial reported comparable 5-year outcomes between TME and CRT-TEM: local recurrence rates were 6.2% vs. 7.4%, distant recurrence rates were 17.3% vs. 12.3%, and overall survival rates were 85.2% vs. 82.7%, with identical disease-free survival rates (88.9%) in both groups [7]. Additionally, interim findings from the STAR-TREC trial, presented at ESTRO 2025 [8, 9], showed that 80% of patients who received long-course CRT and 61% of those who received short-course RT avoided radical surgery at 1-year follow-up. These results support the expanding role of organ-preserving strategies in the management of rectal cancer.

The scenario varies for incidental pT2 adenocarcinomas found after local excision. In such cases, options include salvage surgery, adjuvant (chemo) radiotherapy, or surveillance, guided by pathological risk and quick assessment of possible mesorectal nodal involvement. Even when nodal status is presumed to be N0, caution is necessary. A meta-analysis [10] reported a 28.9% local recurrence rate for pT2 tumours treated with local excision alone, reduced to 14.7% with adjuvant CRT, but still less favourable than the 4% recurrence rate with completion TME [11]. These findings match those of Hu et al. [12], who analysed selected pT1 high-risk and pT2 patients unfit or unwilling to undergo radical surgery: TEM plus adjuvant CRT significantly improved 3-year local recurrence-free survival (93.3% vs 66.6%) and disease-free survival (93.3% vs 63.7%) compared to TEM alone, with local recurrence rates of 5% vs 31%, respectively. CRT became an independent predictor of DFS, although patient numbers were small.

In our series, overall survival after salvage surgery aligned with previous studies, supporting its potential to cure when pT2 disease is found at final pathology. Radiotherapy showed the highest OS and DFS, especially in patients unfit or unwilling to undergo major surgery. These findings emphasise the role of postoperative RT as an effective, organ-preserving alternative in selected patients, particularly when considering the morbidity linked to completion TME. It is well known that TME can cause significant urinary and sexual dysfunction, defecatory issues (anterior resection syndrome), and a high chance of temporary or permanent stoma—especially in low anastomoses or if complications occur [13, 14]. Therefore, avoiding radical resection whilst achieving acceptable oncologic outcomes is a valid treatment aim. However, concerns remain regarding the safety of neoadjuvant RT protocols. The NERATEM pilot study [15], which assessed short-course RT followed by TEM in T1–T2N0 extraperitoneal rectal cancers, was stopped early due to high rates of rectal suture dehiscence (50%) and severe complications, including enterocutaneous fistulas. These results serve as a warning against routine use of short-course RT prior to TEM in early stage disease. In the present series, all patients received long-course RT, without chemotherapy.

Our analysis also revealed poor outcomes amongst patients who received no further treatment despite high-risk pathological features. This group experienced the highest recurrence and mortality rates, supporting guideline recommendations against local excision alone for pT2 tumours [16]. Salvage surgery offered satisfactory OS but marginally lower DFS than radiotherapy, possibly due to selection bias or delayed radical treatment allowing occult micrometastatic progression.

Histopathological features—particularly tumour budding, lymphovascular invasion, and poor differentiation—emerged as possible key prognostic factors, consistent with previous literature. Still, only tumour budding was confirmed as a predictor of both OS and DFS in the Cox proportional hazard regression models multivariate analysis, as previously reported [17, 18]. This adverse marker retained its prognostic influence even amongst patients undergoing salvage surgery, suggesting that tumour biology may ultimately outweigh treatment intensity in determining long-term outcomes. Our multivariate analysis underscores the value of these features in guiding post-excision management and identifying candidates for radicalisation.

Overall, our data support the use of postoperative radiotherapy as a reasonable compromise for patients diagnosed with pT2 tumours only after local excision, especially those who are inoperable or wish to avoid the functional decline associated with radical pelvic surgery. Whilst salvage surgery remains the standard treatment, our results suggest a protective role for adjuvant RT. The ongoing TESAR trial [19] will offer further clarity, comparing adjuvant CRT with completion TME in pT2 patients with intermediate-risk features following local excision. Although neoadjuvant radiotherapy remains standard in many protocols, TESAR aims to determine whether tailored adjuvant strategies with close surveillance and early salvage intervention can provide oncological safety whilst preserving organs. Furthermore, improvements in intraoperative staging of rectal cancer at the time of local excision, such as the use of fluorescence to better characterise tumour histology and invasiveness [20], or sampling of potential sentinel lymph nodes in the mesorectum [21, 22], could significantly alter the perspective on organ-sparing techniques.

This study is limited by its retrospective, single-centre design and modest sample size. The long inclusion period may have introduced variation in techniques and treatment protocols, due to the potential impact of evolving standards of care and imaging modalities across the three-decade study period. Additionally, selection bias cannot be excluded: younger or higher-risk patients may have been preferentially referred for salvage surgery, and delays in radicalisation may have contributed to micrometastatic progression. Moreover, the heterogeneity of treatments prevents any subgroup analysis for the dimension of our dataset. Therefore, prognostic factors for recurrence and survival are likely confounded by the strategy chosen after local excision. Ultimately, systematic collection of postoperative functional outcomes and quality-of-life metrics was not available for this retrospective series. Nonetheless, this cohort represents the most significant single-institution experience explicitly focussed on pT2N0 rectal adenocarcinoma treated with transanal excision, with standardised follow-up and histopathological review.

In conclusion, our findings support a risk-adapted strategy for managing pT2N0 rectal adenocarcinoma following local excision. Whilst salvage surgery remains the standard for high-risk histologic features, adjuvant radiotherapy may offer comparable disease control in carefully selected, non-surgical candidates. Tumour biology—including tumour budding, vascular and perineural invasion, and high-grade histology—should guide postoperative decisions. Local excision alone should be avoided in the presence of high-risk features, and all patients should undergo multidisciplinary evaluation to tailor further management. Future prospective studies are needed to confirm whether radiotherapy combined with detailed pathological risk stratification can safely expand the role of organ-preserving strategies in this setting.
